# Population dynamics of *Brachionus calyciflorus* driven by the associated natural bacterioplankton

**DOI:** 10.3389/fmicb.2022.1076620

**Published:** 2023-01-16

**Authors:** Yongzhi Zhang, Sen Feng, Lingyun Zhu, Meng Li, Xianling Xiang

**Affiliations:** ^1^School of Ecology and Environment, Anhui Normal University, Wuhu, Anhui, China; ^2^Collaborative Innovation Center of Recovery and Reconstruction of Degraded Ecosystem in Wanjiang Basin Co-founded by Anhui Province and Ministry of Education, Wuhu, Anhui, China

**Keywords:** bacterioplankton, associated bacteria, population growth, life history characteristics, high-throughput sequencing, *Brachionus calyciflorus*

## Abstract

Zooplankton provides bacteria with a complex microhabitat richen in organic and inorganic nutrients, and the bacteria community also changes the physiochemical conditions for zooplankton, where the symbiotic relationship between them plays an important role in the nutrient cycle. However, there are few studies on the effect of associated bacteria on the population dynamics of rotifers. In order to make clear their relationships, we reconstructed the associated bacterial community in *Brachionus calyciflorus* culture, and examined the life history and population growth parameters, and analyzed the diversity and community composition of the associated bacteria at different growth stages of *B. calyciflorus*. The results showed that the addition of bacteria from natural water can promote the population growth and asexual reproduction of *B. calyciflorus*, but has no significant effect on sexual reproduction, exhibited by the improvement of its life expectancy at hatching, net reproduction rates and intrinsic growth rate, no significant effects on the generation time and mixis ratio of offspring. It was found that the *B. calyciflorus*-associated bacterial community was mainly composed of Proteobacteria, Bacteroidota, Actinobacteriota, Cyanobacteria and Firmicutes. Through correlation network analysis, the members of Burkholderiales, Pseudomonadales, Micrococcales, Caulobacterales and Bifidobacteriales were the keystone taxa of *B. calyciflorus*-associated bacteria. In addition, the relative abundance of some specific bacteria strains increased as the population density of *B. calyciflorus* increased, such as *Hydrogenophaga*, *Acidovorax*, *Flavobacterium*, *Rheinheimera*, *Novosphingobium* and *Limnobacter*, and their relative abundance increased obviously during the slow and exponential phases of population growth. Meanwhile, the relative abundance of adverse taxa (such as *Elizabethkingia* and Rickettsiales) decreased significantly with the increase in rotifer population density. In conclusion, the closely associated bacteria are not sufficient for the best growth of *B. calyciflorus*, and external bacterioplankton is necessary. Furthermore, the function of keystone and rare taxa is necessary for further exploration. The investigation of the symbiotic relationship between zooplankton-associated bacterial and bacterioplankton communities will contribute to monitoring their roles in freshwater ecosystems, and regulate the population dynamics of the micro-food web.

## 1. Introduction

In freshwater ecosystems, bacterioplankton and metazoan zooplankton, as separate functional taxa, play an important and necessary role in energy flow, matter mineralization and nutrient cycling (Azam and Malfatti, 2007). However, microbial ecologists often overlook zooplankton as potential habitats for aquatic bacteria. Meanwhile, zooplanktonologists tend to focus much more on interactions between zooplankton and their prey or predators (Tang et al., 2010). Thus, the interaction between bacterioplankton and metazoan zooplankton is poorly understood.

Many organisms rely on a complex symbiotic network to perform a range of functions from defense (immunity) to metabolism in the holobiont system (Wang, 2017; Haag, 2018). As a comparison, associated microorganisms are different from symbiotic microbiota. Associated microorganisms and zooplankton are important components of planktonic food webs, and they are usually regarded as independent functional units though inhabiting the same aquatic ecosystem, which does not have a strict symbiotic relationship, but are only indirectly connected through matter cycling and trophic cascades (Tang, 2005; Azam and Malfatti, 2007; Tang et al., 2010). To date, several studies have revealed that zooplankton induces a high abundance of diverse bacterioplankton, which is greater than those in the surrounding water (Freese and Schink, 2011; Peerakietkhajorn et al., 2016). Zooplankton can release and consume amounts of particulate and dissolved organic matter (DOM), which therefore provides a complex microhabitat for bacteria to thrive. Among them, associated bacteria may be optimally positioned to exploit these resources (Carman, 1994; Corte et al., 2018). For example, the exoskeleton and gut lining of zooplankton provide an opportune condition for bacterial attachment (Freese and Schink, 2011; Shoemaker and Moisander, 2017), simultaneously, which may provide refuge for associated bacteria against external hazards (Tang et al., 2011). In addition, some studies demonstrated an active bacteria exchange between the zooplankton and the surrounding waters (Grossart et al., 2010), and they share similar bacterial groups but in different compositions (Shoemaker et al., 2019) because the bacteria composition is highly flexible and strongly affected by the environment (Eckert et al., 2021).

Rotifera is one of the smallest metazoa of which over 2,200 species have been described (Le et al., 2017). It has been proven that the addition of probiotic bacteria to rotifer cultures brings positive effects (Hagiwara et al., 1994; Le et al., 2017). Although rotifers feed on bacteria, they prey on fewer bacteria than algae (Miracle et al., 2014; Contreras-Tapia et al., 2020a), and did not grow on the probiotics as the sole food source (Contreras-Tapia et al., 2020b). Bacterial community composition (BCC) in the habitat has long been recognized to influence rotifer culture stability and population growth (Zink et al., 2013; Turgay et al., 2020). These studies have only analyzed the associated bacterial community, while omitting the symbiotic bacterial community of rotifers (Sakami et al., 2014; Raatz et al., 2018). In addition, many of the studies evinced that crustacean-associated bacteria are often acquired by the horizontal transmission of microbes present in the environment, and different compositions can provide different effects on the host (Callens et al., 2018). Although some studies have been conducted to investigate the associated bacterial composition of specific rotifers (Selmi et al., 2001; Prol-García et al., 2010), it is still not enough to account for the effect of associated bacteria on the population dynamics of rotifers.

The suspension-feeding rotifer *Brachionus calyciflorus* Pallas is a cosmopolitan zooplankton species that reproduces with cyclic parthenogenesis (Jensen and Verschoor, 2004). The factors that have been shown to have an effect on the population dynamics of *B. calyciflorus* include food (Ozdemir, 2009), temperature (Gilbert, 2020), interspecies competition (Zhang et al., 2019), and more. However, few studies focus on the effect of the associated bacteria composition on the population dynamics of *B. calyciflorus* (Contreras-Tapia et al., 2020a).

Recent studies tend to stress that the presence of a microbiome is quite essential for the functioning of zooplankton. Furthermore, microbiomes of zooplankton organisms also tend to select bacteria present in their environment (Tang et al., 2010). With the aim to clarify the relationship between the associated bacteria and population dynamics of *B. calyciflorus*, we examined the life history and population growth parameters, and analyzed the diversity and community composition of the associated bacteria at different growth stages of *B. calyciflorus* by high-throughput sequencing. Ultimately, it is expected to verify the following hypotheses: (1) The closely associated bacteria are not sufficient for the best growth of *B. calyciflorus*, so the bacterioplankton possibly have a promoting effect; (2) The community composition of *B. calyciflorus*-associated bacteria may be closely related to the environmental bacteria in surrounding water; (3) The keystone taxa of *B. calyciflorus*-associated bacteria with vary probably at different stages of growth.

## 2. Materials and methods

### 2.1. Resting eggs isolation, sterilization and hatching

The sediments were collected from Lake Jinghu (31°19′45″N, 118°22′29″E) in Wuhu city (1 cm surface layer of pond mud). We collected the distilled water (RC-ZLA-20 l, Beijing Ruicheng Yongchuang Technology Co., Ltd.), we sterilize it at 121°C for 30 min using an autoclave (GR60O, Zhiwei (Xiamen) Instrument Co., Ltd.) to make sterile water. Rotifer resting eggs in the sediments were separated with a sugar flotation method (Onbé, 1978; Gómez and Carvalho, 2000). We placed the separated resting eggs in test tubes containing sucrose solution (1 kg sucrose dissolved in 1 l distilled water) and centrifuged them at 3000 r/min for 3 min, and the supernatant was filtered through a 30 μm Nytal mesh and rinsed with sterile water. The filtrate was resuspended in sterile water and *B. calyciflorus* resting eggs were picked out under a stereoscope (SMZ168-BLED, Motic China group co., Ltd.). After collection, the resting eggs of *B. calyciflorus* were sterilized according to the protocol by Douillet (1998, 2000). First, the resting eggs were rinsed 3–5 times using a micropipette in a beaker filled with sterile water, transferred to a beaker containing 0.5% sodium hypochlorite solution (ready-to-use) and immersed for 3 min, then washed in the sterile water for 3–5 times again. In order to verify the sterility of resting eggs, some resting eggs were randomly inoculated in Luria-Bertani media and cultured at 37°C for 2–3 days. After a contamination had been detected, this batch of resting eggs was discarded fully, and the disinfection process was repeated to obtain resting eggs without a bacterial growth in the control culture media. However, only external cultivable bacteria were removed from the surface of the rotifer eggs due to the sodium hypochlorite treatment. Finally, the decontaminated resting eggs were introduced into an 8 mL glass jar with 5 mL sterile EPA media for monoclonal culture. The single clone with the highest vitality was selected for the experiment. Hatching was carried out in a constant temperature illuminated incubator (PGX-350C, Ningbo Saifu Experimental Instrument Co., Ltd.) at 25 ± 1°C with a light intensity of 2000 Lux and light–dark period of 14 l:10D regime, and all experimental operations and observations were performed quickly in Super Clean Workbench (SW-CJ-1FD, Suzhou Saihongtai Purification Technology Co., Ltd.). Generally, the resting eggs hatched within 24–36 h.

### 2.2. Pre-culture of decontaminated rotifers

In this experiment, three kinds of rotifer culture experimental treatments were prepared as follows:

Sterile EPA media (SE): The EPA media was sterilized at 121°C for 20 min, which contained 96 mg NaHCO_3_, 60 mg CaSO_4_, 60 mg MgSO_4_·7H_2_O and 4 mg KCl per liter of distilled water (pH 7.4–7.8; Peltier and Weber, 1985).

Sterile EPA culture media with rotifer-associated bacteria (SEB): 1L of fresh water was sampled every day from Lake Jinghu in Wuhu City, and filtered by qualitative filter paper (moderate speed, pore size 30 ∼ 50 μm) to remove zooplankton and impurities without blocking the passage of microorganisms, then filtered again with Millipore filter membrane (0.22 μm, Aquo-system, Cat No. F513134, Sangon Biotech (Shanghai) Co., Ltd.), to obtain bacterioplankton. Finally, the filter membrane was washed with an equal amount (1 l) of sterile EPA, and the mixture was used as the culture media. The media needs to be resampled daily for preparation until the end of the experiment.

Natural lake water (NW): The fresh water was sampled every day from Lake Jinghu, and filtered by qualitative filter paper described above to remove zooplankton and impurities, then used as the culture media.

Before starting the experiments, the rotifer population was precultured in the three kinds of rotifers media (SE, SEB and NW) at 25 ± 1°C with a light intensity of 2000 Lux and a light–dark period of 14 l:10D regime for at least 2 weeks to minimize maternal effects. The algae *Tetradesmus obliquus* purchased from the Freshwater Algae Culture Collection at the Institute of Hydrobiology (FACHBcollection), were semi-continuously cultured in HB-4 media (Andersen, 2005) with the 16 l:8D photoperiod of 3,000 Lux fluorescent light and 28 ± 1°C in illumination incubator. The algae were used as food for rotifer with a density of 2 × 10^6^cells/mL, and the culture media was renewed every 24 h and simultaneously the fresh food was supplied. The rotifer was kept in an exponential growth period until it was used. During the experimental operation, bacteria in the food alga and inside the resting eggs will inevitably infect the rotifers culture, but the effect is the same for the three treatment groups. We performed high-throughput sequencing of associated bacteria with *T. obliquus* as food for rotifers, as a reference.

### 2.3. Life table demography

In this experiment, the pre-cultured *B. calyciflorus* with amictic eggs was transferred into a sterile beaker with EPA media and algae, and the neonates born within 2 h were selected for an experiment. For each treatment, 10 neonates were introduced into 8 mL glass beakers containing 5 mL culture media with an algal density of 2.0 × 10^6^ cells/mL. Each treatment consisted of three replicates. The operation was conducted in Super Clean Workbench, then the containers were put in a bioclimatic chamber with the same conditions as pre-culture. During the experiment, the number of surviving mothers and juveniles of rotifers was observed and recorded every 8 h. Meanwhile, we suspended the culture for preventing the deposition of food in the experiment, and the larvae were transferred into a new culture plate, and continued to be cultured under the same conditions until the female type was determined after carrying eggs. The culture media was renewed every 24 h and simultaneously fresh food was supplied. After every individual in each cohort died, the experiment ended.

Based on the collected data, the life expectancy at hatching (*e*_0_), the net reproductive rate (*R*_0_) and generation time (*T*) were calculated with Equations 1–3, respectively (Krebs, 1985). The intrinsic rate of population increases (*r_m_*) was first approximated using: r-rough = ln*R*_0_/*T*. For the final calculation, we solved it with Equation 4.


(1) $$e_{x}=\frac{T_{x}}{n_{x}}$$

(2) $$R_{0}=\overset{\infty }{\underset{0}{\sum }}l_{x}m_{x}$$

(3) $$T=\frac{\sum xl_{x}m_{x}}{R_{0}}$$

(4) $$\overset{n}{\underset{x=0}{\sum }}e^{-rx}l_{x}m_{x}=1$$

Where *T_x_* is the sum of the remaining lifetime of all individuals in this age group, *n_x_* is the number of alive rotifers at time *x*; the age-specific survivorship (*l_x_*) is the percentage of surviving individuals in age group *x*; the age-specific fecundity (*m_x_*) is the average number of female offspring reproduced by each individual in age group *x*. Mixis ratios (*MR*) was the proportion of sexual rotifer offspring.

### 2.4. Population growth experiment

From the pre-culture, 90 neonates (less than 2 h old) were collected and placed equally into 9 glass jars (3 culture media × 3 replicates) containing 5 mL of EPA media with 2.0 × 10^6^ cells/mL of algae food. The rotifers were maintained in a 25 ± 1°C bioclimatic chamber with a light intensity of 2000 Lux and a light–dark period of 14 l:10D regime, and counted all rotifers once every day in Super Clean Workbench in the first few days, while counted by random sampling when the population is large. The culture media was renewed every 24 h and simultaneously fresh food was supplied. The experiment ended 2–3 days after the population density of all treatment groups started to decline.

According to the recorded data, the time interval was selected from the second day to the sixth day, and the population growth rate is calculated with reference to Krebs (1985) equation:


$$r=\left(\ln N_{t}-\ln N_{o}\right)/t$$

Where *N*_0_ is the inoculation density of rotifer at the beginning of the experiment (2 ind./mL); *N_t_* is the population density of rotifer at time *t* in days.

### 2.5. Next, generation sequencing of 16S rRNA gene from associated bacterial communities

Another 12 replicates were established during the population growth experiment, and the experimental conditions were the same as those of the SE, SEB and NW groups, respectively. *B. calyciflorus* was cultured with the same inoculum density (2 ind./mL) and culture conditions. For any group, four replicates were randomly selected at the stage of 0, 3, 6 and 9 days (corresponding to the periods of start, slow growth, exponential growth and decline) to estimate the *B. calyciflorus*-associated bacterial community. It should be noted that destructive sampling was used in the experiment. Because of the small size of *B. calyciflorus*, to ensure sufficient sample size for sequencing, all rotifer individuals in glass jar should be transferred to sterile containers, washed with sterile distilled water 3–5 times to remove bacteria in culture media and bacteria involved in loose associations with the rotifers body surface, then transferred to 1.5 mL sterile Eppendorf tube and stored at-20°C, waiting for further 16S rRNA sequencing.

Genomic DNA of *B. calyciflorus*-associated bacterial communities was extracted from samples using the E.Z.N.A.® Soil DNA Kit (Omega Bio-tek, Norcross, GA, United States). After the DNA was purified, the DNA was determined by 1% agarose gel electrophoresis, and the DNA concentration and purity were inspected by NanoDrop 2000 UV–Vis spectrophotometer (Thermo Scientific, Wilmington, United States). The V3-V4 hypervariable region of bacterial 16S rRNA gene was amplified by PCR thermal cycling apparatus (GeneAmp® 9,700, ABI, United States) using primers 338F (5’-ACTCCTACGGGAGGCAGCAG-3′) and 806R (5’-GGACTACHVGGGTWTCTAAT-3′). PCR amplification was carried out using TransStart Fastpfu DNA Polymerase (TransGen AP221-02) in a 20 μl reaction system, and amplification parameters were as follows: the initial denaturation at 95°C lasted for 3 min, then denaturation at 95°C for 30 s, annealing at 55°C for 30 s, and extension at 72°C for 45 s, 27 cycles in total, with a single extension at 72°C for 10 min and termination at 10°C. The PCR product was extracted from a 2% agarose gel, purified using the AxyPrep DNA Gel Extraction Kit (AxyPrep Biosciences, Union City, United States) and quantified using a Quantus™ fluorometer (Promega, United States). According to the standard protocol of Majorbio BioPharm Technology Co., (Shanghai, China), amplicons were prepared, purified, pooled in equimolar concentrations, and sequenced in a 2 × 300 paired-end run using NEXTFLEX® Rapid DNA-Seq Kit (Bioo Scientific, United States) on an Illumina MiSeq (PE300) platform (Illumina, San Diego, United States).

### 2.6. Bioinformatic and statistical treatment of data

In this study, data analysis and mapping are carried out with the help of the Majorbio Online Cloud Platform.^1^ UPARSE (version 7.1^2^) was used to cluster operational taxons (OTUs) with 97% similarity cut-off value, and chimeric sequences were identified and deleted. The classification of each OTU representative sequence after removed singletons were analyzed against the Silva database (version 138^3^) using a 70% confidence threshold using the RDP classifier (version 2.11^4^). Meanwhile, the chloroplasts need to be removed, and then the OTU abundances were normalized by using standard sequence numbers that corresponded to the samples with the least sequences. The subsequent alpha diversity and beta diversity analyzes were performed based on the normalized data. Using Qiime (version 1.9.1^5^) to calculate alpha diversity index under different random sampling. Using R language (version 3.3.1) vegan package to complete NMDS analysis, venn diagram, species composition analysis, heatmap diagram, and using R language stats package and Python scipy package to complete interspecies difference test and correlation. Further, using the R language randomForest package and Python networkx package to complete model prediction (Random Forest and Network) analysis and mapping, respectively.

All data were analyzed by using SPSS 22.0 and expressed as Mean ± SE (standard error). The one-sample Kolmogorov–Smirnov procedure and Levene’s test were used to test the data for normality and homogeneity of variances, respectively. One-way analysis of variance (ANOVA) was conducted to identify the significant effect of different media on the variables of population growth and life history. For the parameters with significant effects, multiple comparisons were conducted using Student–Newman-Keulsa (SNK) to identify which groups were significantly different among treatments. In the process of variance analysis, the data that do not conform to the normal distribution were adjusted logarithmically. For data with homogenous variance, Duncan’s method was selected to compare the differences among groups, and the data with uneven variance were compared by the Games-Howell method.

## 3. Results

### 3.1. Life history parameters

**Figure 1:**
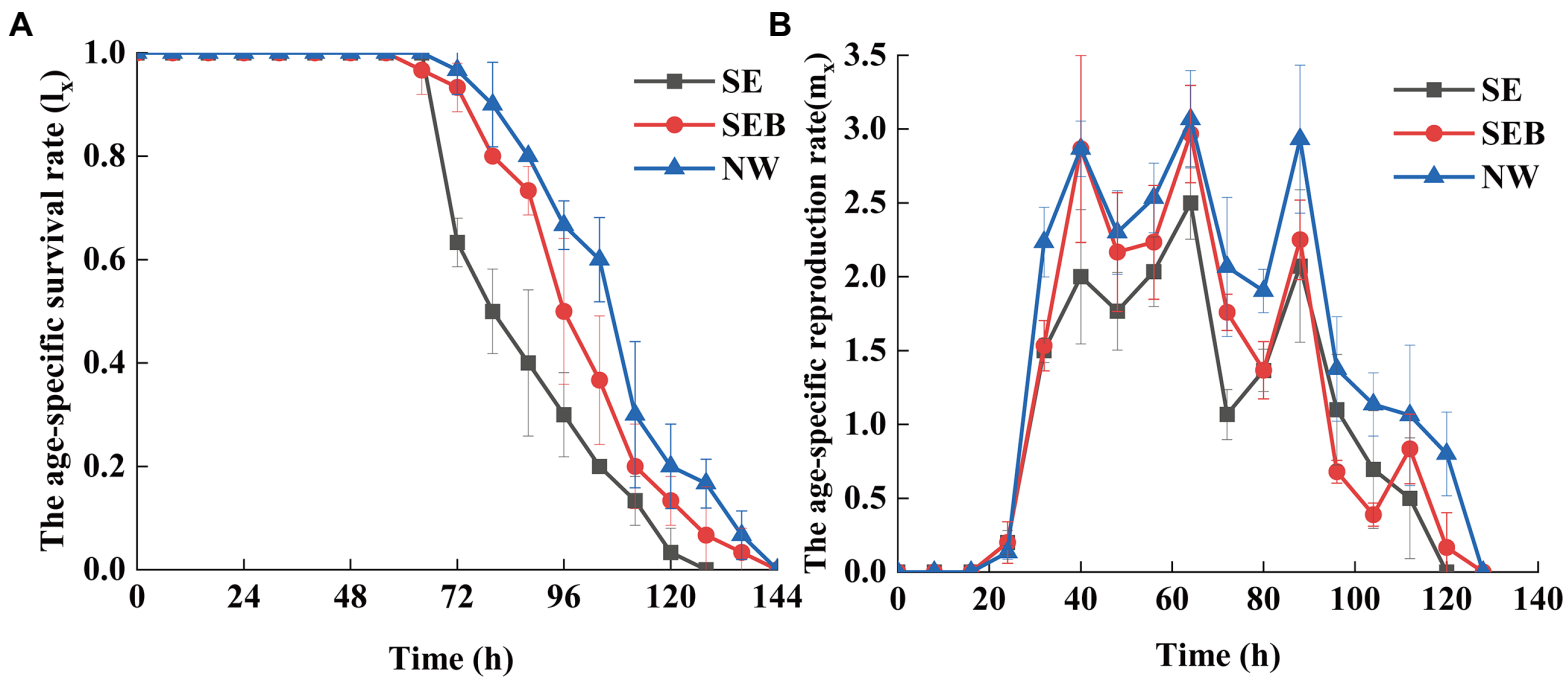
The age-specific survival and reproduction curves of *Brachionus calyciflorus* in different treatmens. **(A)** The age-specific survival rate. **(B)** The age-specific reproduction rate.

The existence of a bacterial community in the culture media improved the survival rate and reproduction rate of *B. calyciflorus* (Figure 1). According to the age-specific survival curve (Figure 1A), none of *B. calyciflorus* experienced death in the first 64 h for the three groups, and the survival rate of the SEB group began to decline at 64 h, while that of the other groups began to decline at 72 h. Besides, all rotifers in the SE group died at 128 h, and their death rate was much fast, while all the SEB and NW groups died until 144 h. According to the reproductive curve of a specific age (Figure 1B), the three treatment groups began to reproduce after 24 h, and then the reproductive rate stabilized and began to decline after 88 h. The difference was that the reproduction rate of rotifers in the SE group was lower than the other groups (Figure 1B).

ANOVA analyzes on different life history parameters showed that culture media affected three life history components (*e*_0_, *R*_0_ and *r_m_*) significantly, but not on the generation time (*T*) and mixis ratio (*MR*) (Table 1). Specifically, there was no significant difference in life expectancy at hatching (*e*_0_) of *B. calyciflorus* between SEB and NW groups which included bacteria in the culture, but it was significantly higher than the SE group (*p* < 0.05; Table 2). The net reproductive rates (*R*_0_) and intrinsic growth rate (*r*_m_) in the NW group were significantly higher than the SE groups, while the SEB group was not significantly different from the two groups (Table 2). For the generation time (*T*) and mixis ratio of offspring (*MR*), there was no significant difference among the three groups (Table 2).

**Table 1:** The one-way ANOVA of life history and population growth parameters in *B. calyciflorus*.

Parameters		Sum of squares	df	Mean square	*F*	Sig.
*e*_0_	Between	567.182	2	283.591	15.578	0.004
	Within	109.227	6	18.204		
	Total	676.409	8			
*R*_0_	Between	110.116	2	55.058	10.581	0.011
	Within	31.222	6	5.204		
	Total	141.338	8			
*T*	Between	59.234	2	29.617	4.231	0.071
	Within	42.004	6	7.001		
	Total	101.238	8			
*r_m_*	Between	0.000	2	0.000	5.701	0.041
	Within	0.000	6	0.000		
	Total	0.000	8			
*MR*	Between	0.000	2	0.000	0.238	0.795
	Within	0.003	6	0.001		
	Total	0.004	8			
*r*	Between	0.039	2	0.020	6.672	0.030
	Within	0.018	6	0.003		
	Total	0.057	8			
*D*_max_	Between	30094.649	2	15047.324	43.108	0.000
	Within	2094.347	6	349.058		
	Total	32188.996	8			

There is no significant difference in multiple comparisons between groups with the same letter indicating the same row. *e*_0_, life expectancy at hatching (h); *R*_0_, net reproduction rates (offspring female_−1_ lifespan_−1_); *T*, generation time (h); rm, intrinsic growth rate (offspring female_−1_ lifespan_−1_); *M*R, mixis ratio of offspring; *r*, population growth rate; *D*max, maximum density. Shown are the mean ± standard error values based on three replicates.

**Table 2:** The multiple comparisons of life history and population growth parameters in *B. calyciflorus* among different treatments.

Parameters	SE	SEB	NW
*e*_0_	85.60 ± 2.85^b^	97.87 ± 3.60^a^	104.80 ± 3.92^a^
*R*_0_	12.74 ± 1.31^b^	16.93 ± 0.70^ab^	21.31 ± 2.86^a^
*T*	56.56 ± 2.04^a^	59.13 ± 2.76^a^	62.81 ± 1.50^a^
*r_m_*	0.0513 ± 0.0019^b^	0.0558 ± 0.0027^ab^	0.0589 ± 0.0021^a^
*MR*	0.02 ± 0.01^a^	0.04 ± 0.03^a^	0.03 ± 0.01^a^
*r*	0.54 ± 0.01^b^	0.58 ± 0.02^b^	0.70 ± 0.07^a^
*D*_max_	113.20 ± 7.38^c^	150.80 ± 16.17^b^	250.30 ± 19.55^a^

Different lowercase letters in the same line indicate significant differences.

### 3.2. Population growth

**Figure 2:**
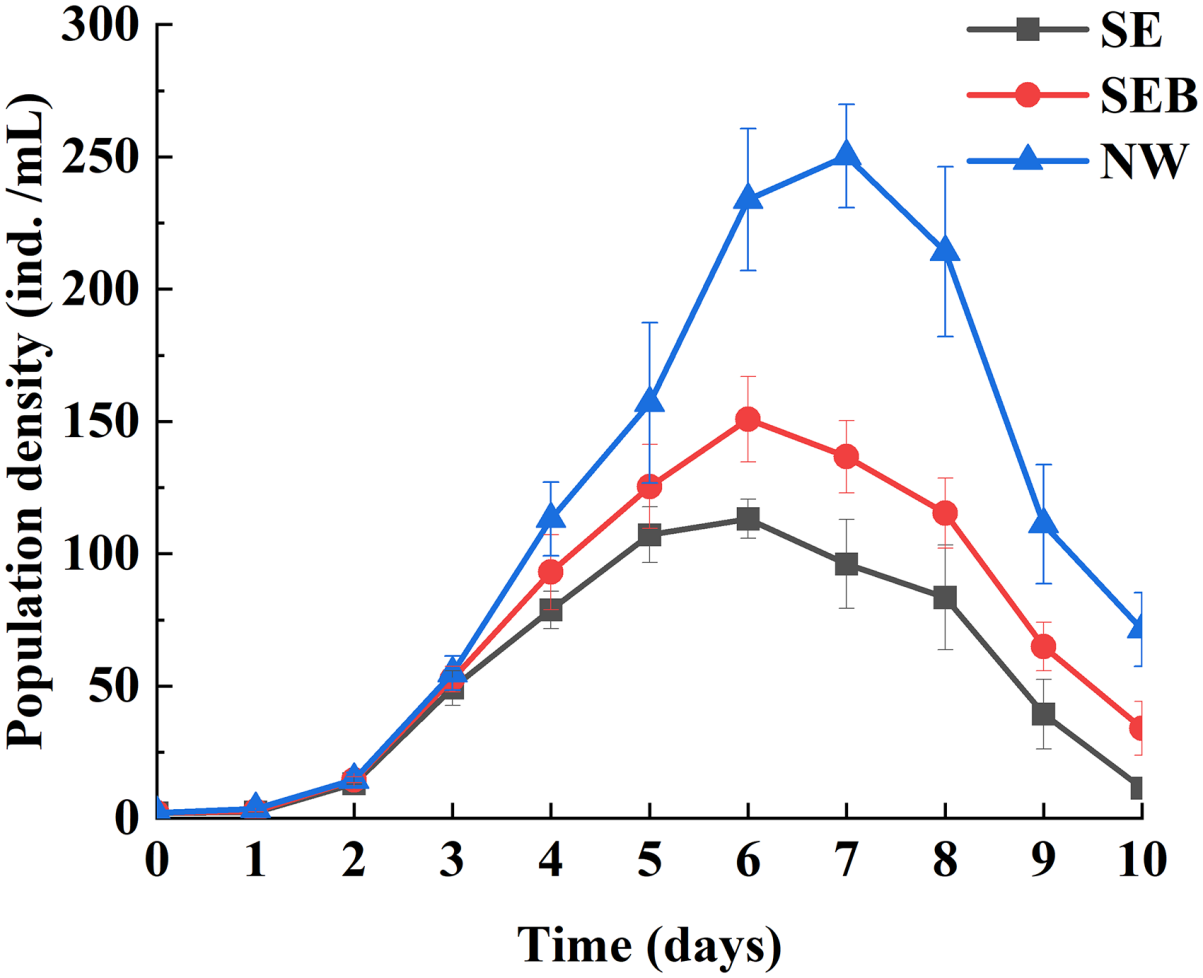
The population density in *B. calyciflorus* under different treatments.

One-way ANOVA analysis showed that the population growth rate (*r*) and the maximal population density (*D*_max_) were affected significantly by the culture media (Table 1, *p* < 0.05). The population density of *B. calyciflorus* did not differentiate among the three groups in the first 3 days (Figure 2) and then increased at different speeds. The population density of the NW group began to decrease after reaching the peak value (250.30 ± 19.55 ind./mL) on the seventh day, and the other two groups (SE and SEB) reached the density peaks (113.20 ± 7.38 and 150.80 ± 16.17 ind./mL) on the sixth day (Figure 2). Among them, the maximum population density of rotifer cultured in the nature water (NW) was significantly higher than that in the SEB media, and the density in the sterile treated group (SE) was significantly lowest by multiple comparisons (Table 2). In terms of population growth rate (*r*), the NW group was significantly higher than the other two groups, and there was no significant difference between these two groups (Table 2).

### 3.3. Alpha and beta diversity of *Brachionus calyciflorus*-associated bacteria community

**Figure 3:**
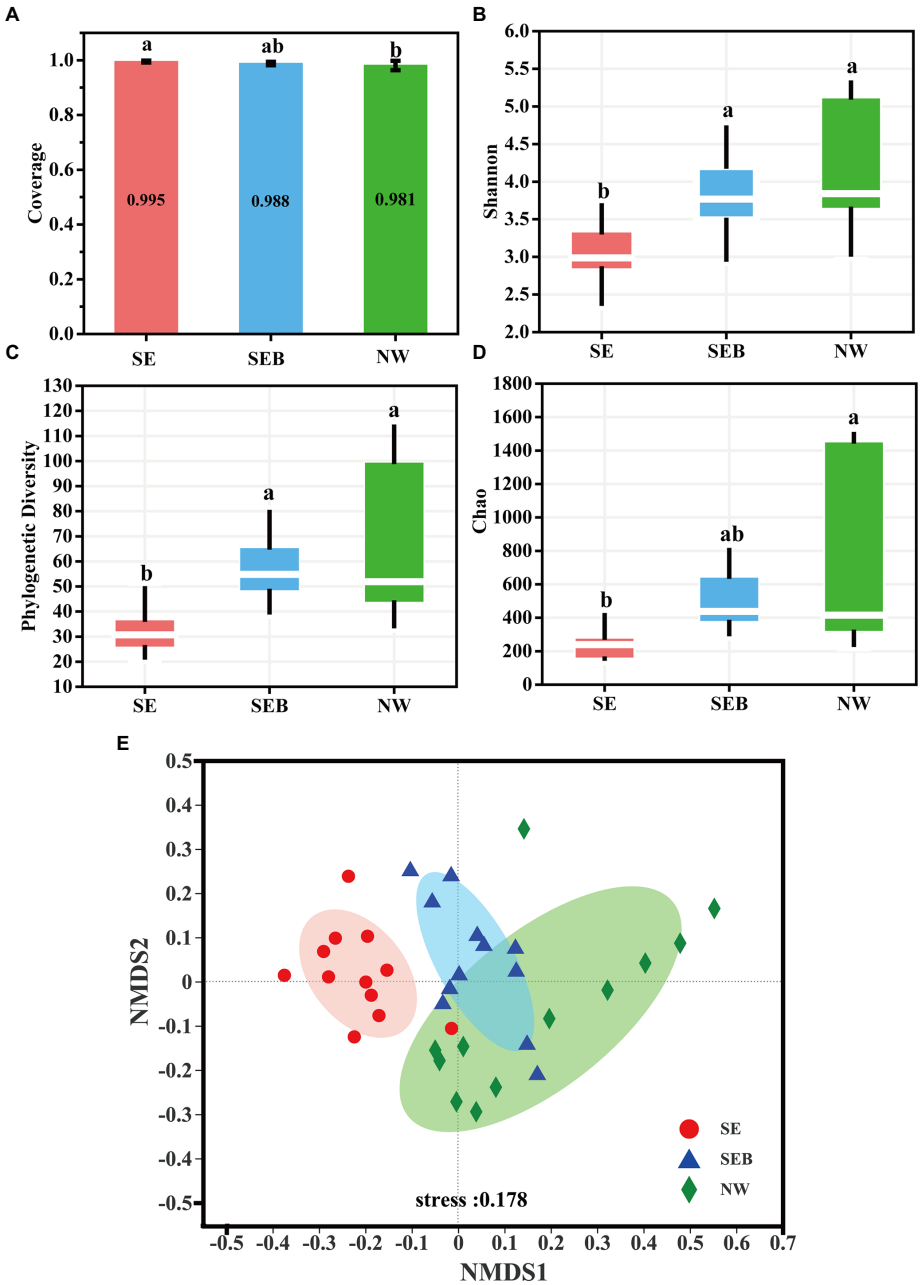
Alpha and beta diversity of *B. calyciflorus*-associated bacterial communities. **(A)** Coverage evenness index. **(B)** Shannon diversity index. **(C)** Phylogenetic index Pd. **(D)** Chao species richness. The bottom and top of the box represent the first and third quartiles, while the thick horizontal line represents the median. In the figure, the same lowercase letter indicates that the difference is not significant, while different letters indicate that the difference is significant (based on wilcoxon rank-sum test). **(E)** Non-metric multidimensional scale analysis (NMDS) based on bray-curtis distance. The inter-group difference test was based on ANOSIM. Points with different colors or shapes represent different groups of samples, and the closer the two sample points are, the more similar the species composition of the two samples is. Horizontal and vertical coordinates represent relative distance, which has no practical significance.

The sequencing Coverage of the three groups of samples is over 98%, indicating that the sequencing depth is enough to cover most bacteria, including rare species (Figure 3A; Supplementary Table S1). There was no significant difference in the Shannon diversity index and phylogenetic diversity index (PD) of the *B. calyciflorus*-associated bacteria between the SEB and NW groups containing natural water bacteria community, but they were significantly higher than the SE group without bacteria (*p* < 0.05; Figures 3B,C). The species richness (Chao) of the NW group was significantly higher than that of the SE group (*p* < 0.05), while there was no significant difference between the SEB group and the other two groups (Figure 3D). Therefore, the consistency of the diversity index indicates that the existence of bacteria in the culture media environment has a significant influence on the composition of *B. calyciflorus*-associated bacteria.

At the genus level, the non-metric multidimensional scaling (NMDS) based on Bray-Curtis dissimilarities showed the aggregation of different repeats and sampling stages. The results showed that the SE treatment was significantly different from the SEB and NW groups (*p* = 0.001), but there was no significant difference between the SEB and NW treatments (Figure 3E). According to Bray–Curtis dissimilarity, the rotifer-associated bacteria communities from natural water (SEB and NW) were far away from the sterile culture media (SE) (Figure 3E).

### 3.4. Composition and differences of *Brachionus calyciflorus*-associated bacterial communities

**Figure 4:**
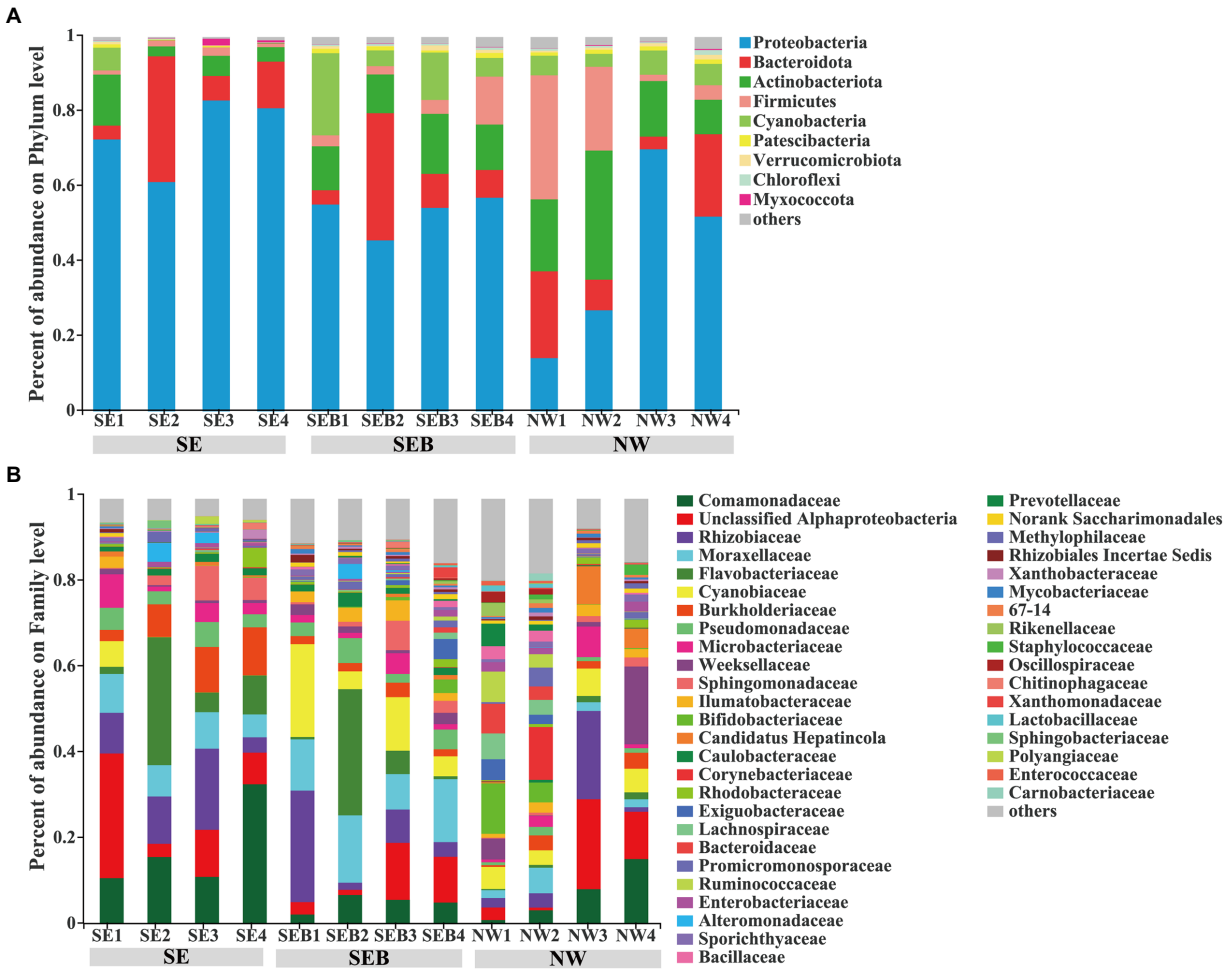
Composition of *B. calyciflorus*-associated bacterial communities. **(A)** at phylum level, **(B)** at family level. The abscissa is the name of the sample, the ordinate is the proportion of the species in the sample, the columns of different colors represent different species, and the length of the columns represents the proportion of the species.

At the phylum level, Proteobacteria (56.14 ± 19.51%) had the highest relative abundance among the three treatment groups, and the order of relative abundance was Bacteroidota (13.94 ± 10.85%), Actinobacteriota (12.80 ± 8.09%), Cyanobacteria (5.90 ± 5.89%) and Firmicutes (7.38 ± 9.85%, Figure 4A). At the family level, the bacterial composition of the SE group is Comamonadaceae (17.46 ± 9.04%), unclassified Alphaproteobacteria (12.77 ± 10.00%), Flavobacteriaceae (11.41 ± 11.14%) and Rhizobiaceae (10.86 ± 5.53%), accounting for more than 50% of the total relative abundance (Figure 4B). In the SEB group, the relative abundance of Moraxellaceae (12.79 ± 2.91%) increased, followed by Cyanobiaceae (10.85 ± 7.19%), Rhizobiaceae (9.82 ± 9.76%), Flavobacteriaceae (9.01 ± 12.08%) and unclassified Alphaproteobacteria (7.08 ± 5.14%, Figure 4B). In the NW group, the order was unclassified Alphaproteobacteria (8.94 ± 8.10%), Rhizobiaceae (6.88 ± 8.08%), Comamonadaceae (6.71 ± 5.51) Weeksellaceae (6.22 ± 7.22%), Cyanobiaceae (5.17 ± 1.09%), and Bifidobacteriaceae (4.39 ± 4.65%; Figure 4B). Under the introduction of the environmental bacteria into culture media, the relative abundance of Comamonadaceae decreased, while the relative abundance of Cyanobiaceae, Bifidobacteriaceae and Weeksellaceae increased, and the relative abundance of unclassified Alphaproteobacteria and Rhizobiaceae did not change significantly (Figure 4B). In addition, we analyzed the bacterial communities associated with algae as food for rotifers. It consisted mainly of Proteobacteria and Bacteroidetes (Supplementary Table S2), and *Brevundimonas* and *Hydrogenophaga* had the highest relative abundance at the genus level (Supplementary Table S3).

**Figure 5:**
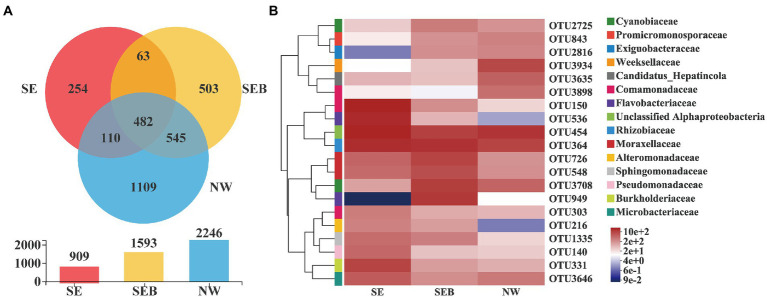
Distribution of OTUs in different treatment groups. **(A)** Venn diagram of OTUs distribution in different treatment groups. Different colors represent different groupings, the numbers of overlapping parts represent the number of species common to multiple groupings, and the numbers of non-overlapping parts represent the number of species unique to corresponding groupings. The bar chart below shows OUTs distribution of each of the three groups. **(B)** Heatmap of the top20 dominant OTUs in different treatment groups. The abscissa is the group name, and the ordinate is the species name. The abundance changes of different species in the sample are displayed through the color gradient of color blocks. On the right side of the figure, the lower side is the value represented by the color gradient, and the upper color level represents the family to which OUT belongs.

The total number of OTUs was 3,309 in the three treatments, and 3,066 after chloroplast elimination, and it was 74 after more than 1% relative abundance filtration. Venn diagram showed that the number of specific OTUs in each group was 254 (SE), 503 (SEB) and 1,109 (NW), respectively, while there were 482 similar OTUs (15.72; Figure 5A). Among the three treatment groups, the OTUs of unclassified Alphaproteobacteria have the highest dominance, followed by Rhizobiaceae, Moraxellaceae, Comamonadaceae, Flavobacteriaceae, Cyanobiaceae and Burkholderiaceae (Figure 5B). In the SEB and NW groups, the relative abundance of OTU150 (Comamonadaceae), OTU536 (Flavobacteriaceae) and OTU331 (Burkholderiaceae) decreased significantly, while the relative abundance of OTU3708 (Cyanobiaceae) and OTU2816 (Exiguobacteriaceae) increased significantly (Figure 5B).

**Figure 6:**
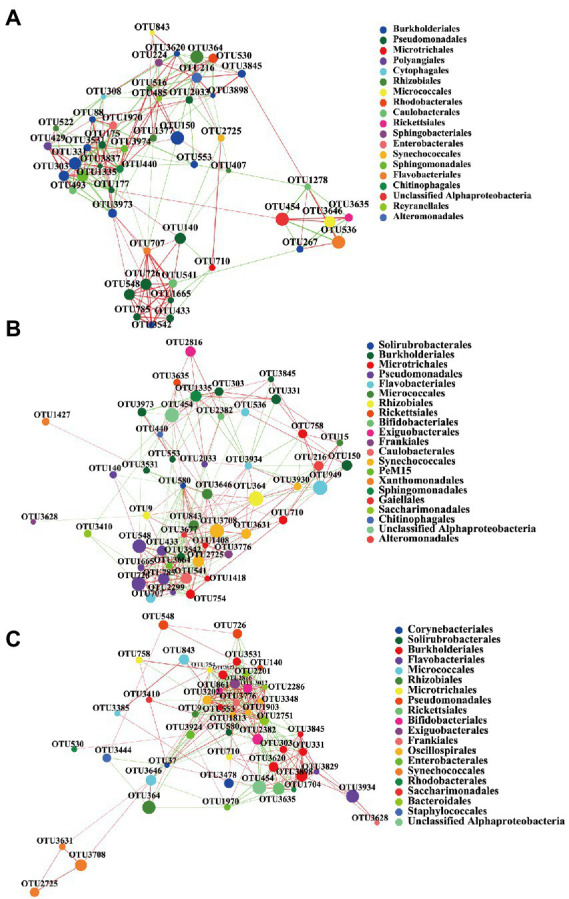
The co-occurrence network of three groups of *B. calyciflorus*-associated bacterial communities based on correlation analysis. **(A)** SE Group, **(B)** SEB Group, **(C)** NW Group. A connection stands for a strong and significant (Spearman’s *p* > 0.5, *p* < 0.05) correlation. The nodes represent unique OTUs and colored according to order. Green edges represent positive correlations and red edges represent negative correlations. Node size is proportional to the betweenness centrality of each OTU, and edge thickness is proportional to the weight of each correlation.

In this study, the complexity of the *B. calyciflorus*-associated bacterial community was evaluated by correlation network analysis, and its potential keystone taxa were inferred. The top 50 dominant taxa of relative abundance at the order level were selected for the network analysis, the correlation coefficients were calculated by spearman, and the data with correlation coefficients greater than 0.5 and *p*-values less than 0.05 were retained for drawing. The highest degree and closeness centrality, and the lowest betweenness centrality can be used to identify keystone taxa together, and their combined scores should be used as thresholds for defining keystone taxa in microbial communities (Banerjee et al., 2018). Therefore, we selected the values that can screen out the top 20 of the three coefficients as thresholds (Banerjee et al., 2019; Zheng et al., 2021). For the SE group, OTUs with an average degree higher than 0.19, closeness centrality higher than 0.43 and betweenness centrality lower than 0.02 were selected as the keystone taxa. For the SEB group, OTUs with an average degree higher than 0.22, closeness centrality higher than 0.50 and betweenness centrality lower than 0.02 were selected as the keystone taxa. For the NW group, OTUs with an average degree higher than 0.27, closeness centrality higher than 0.51 and betweenness centrality lower than 0.02 were selected as the keystone taxa. The complexity of the network structure of the three groups was significantly different. Compared with the SE and SEB groups, the correlation network of the NW group was more complex, with the highest average degree (11.83), average clustering (0.62), and network diameter (6) (Table 3; Figure 6). The network structure of the NW group consists of 10 keystone taxa, which were mainly composed of Exiguobacterales, Solirubrobacterales, Oscillospirales, Burkholderiales, Bifidobacteriales, Bacteroidales, Frankiales, and Microtrichales. However, there were only five keystone taxa in the network structure of the SE and SEB groups, of which the SE group was mainly Caulobacterales, Burkholderiales and Pseudomonadales, while the SEB group was Pseudomonadales, Micrococcales and Caulobacterales. Although the keystone taxa of the three groups of network structures had similar orders, the OTU at the order level is different.

**Table 3:** The network parameters of three treatment groups.

Groups	Avg. degree	Avg. clustering	Diameter	Avg. shortest path length
SE	8.50	0.59	4	2.40
SEB	10.53	0.55	4	2.13
NW	11.83	0.62	6	2.18

### 3.5. Composition dynamics of *Brachionus calyciflorus*-associated bacterial community at different growth stages

**Figure 7:**
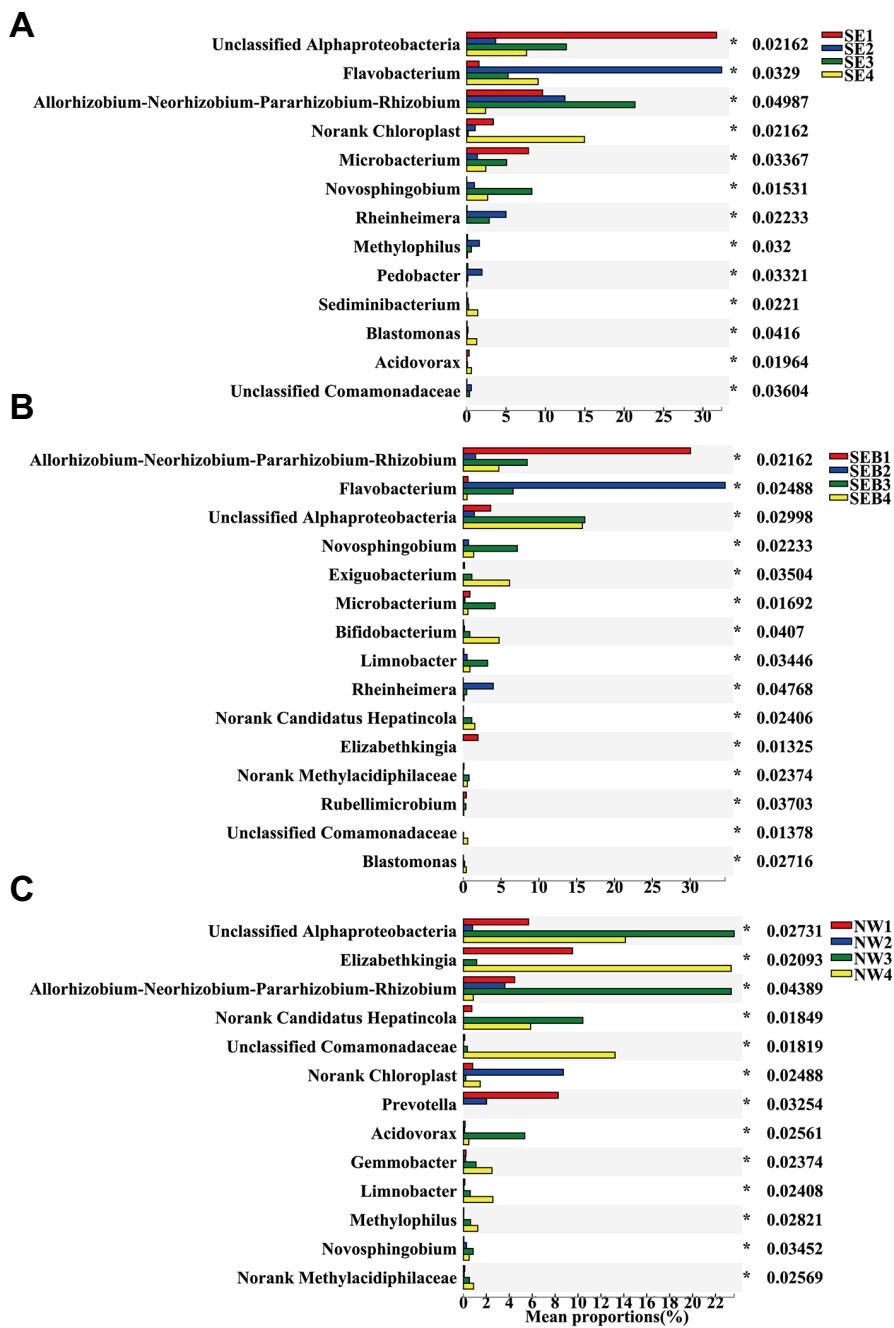
Differences in the community composition of *B. calyciflorus*-associated bacteria at different growth stages of each treatment groups. **(A)** SE Group. **(B)** SEB Group. **(C)** NW Group. Only the genera with significant differences in the top 15 relative abundance were shown. The Y axis represents the species name at a certain taxonomic level, the X axis represents the average relative abundance in different groups of species, and the columns with different colors represent different groups. Based on Kruskal-Wallis rank sum test *p* < 0.05.

The results showed that the relative abundance of *B. calyciflorus*-associated bacteria varied in different periods of rotifer population growth. The relative abundance of *Flavobacterium*, *Novosphingobium*, *Hydrogenophaga* and *Rheinheimera* all increased obviously from the slow growth period to the exponential period and then decreased in the decline period (Figure 7). The relative abundance of *Elizabethkingia* decreased obviously in both slow and exponential growth periods (Figure 7). The relative abundance of unclassified Comamonadaceae and *Limnobacter* increased significantly in the slow and exponential growth period of the SE and SEB treatment groups, while increasing in the decline period of the NW group (Figure 7). The relative abundance of *Acidovorax* raised in the exponential period of the NW group (Figure 7C). The relative abundance of unclassified *Alphaproteobacteria* was significantly lower in the decline period than in the exponential period in the three groups (Figure 7). *Bifidobacterium* increased gradually with the population growth in SE and SEB groups while decreasing gradually in the NW group. *Allorhizobium-Neorhizobium-Pararhizobium-Rhizobium* and *Acinetobacter* showed no obvious change trend among the treatment groups (Figure 7).

### 3.6. Correlation analysis between indicator bacteria and population dynamics in *Brachionus calyciflorus*

**Figure 8:**
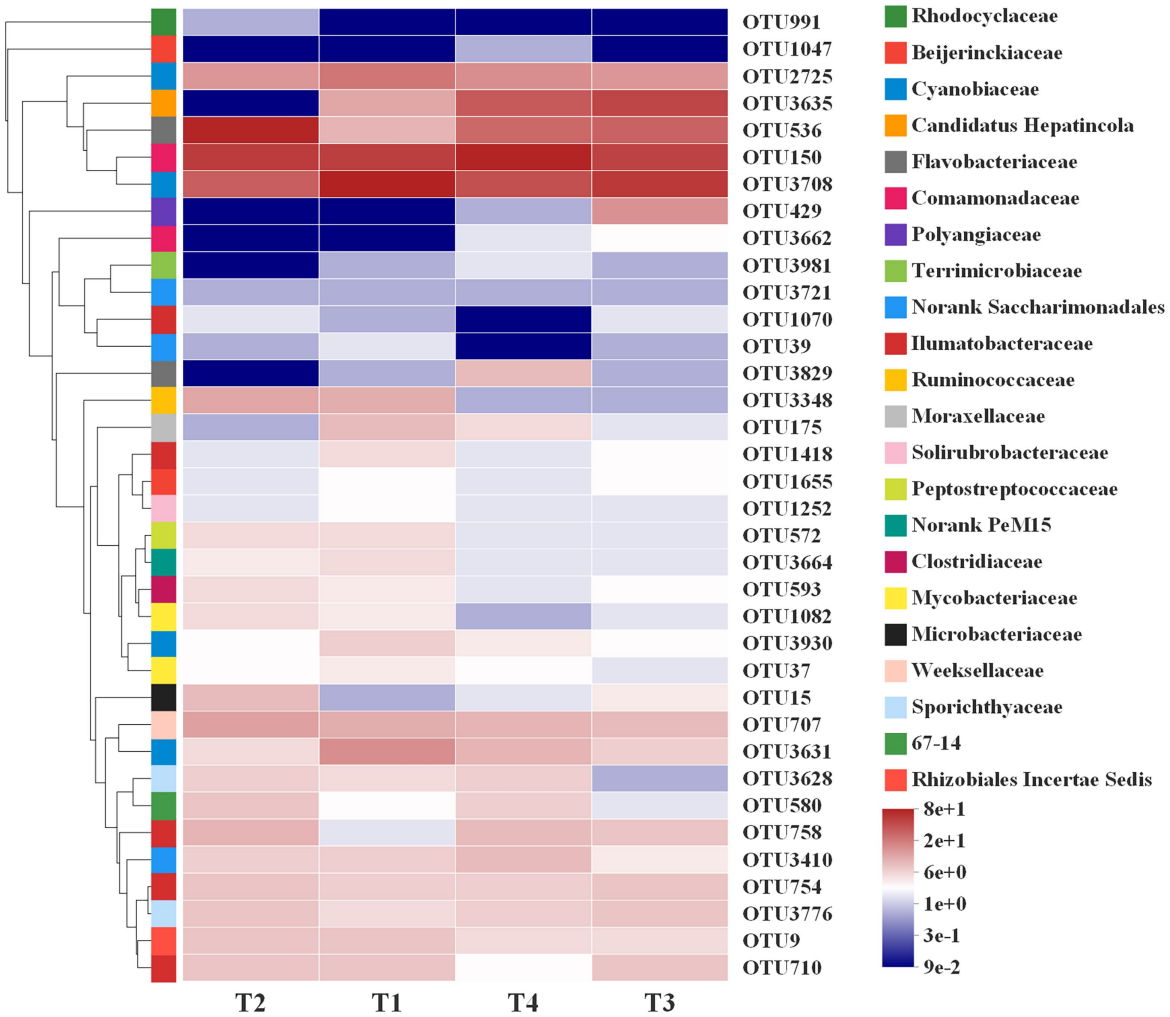
Relative abundance of the first 36 indicator bacteria in different growth stages (T1, T2, T3, and T4) in *B. calyciflorus* based on random forests regression model. The abscissa is the group name, and the ordinate is the species name. The abundance changes of different species in the sample are displayed through the color gradient of color blocks. On the right side of the figure, the lower side is the value represented by the color gradient, and the upper color level represents the family to which OUT belongs.

The random forests regression model was used to explore the responses of the indicator bacteria to rotifer population density at different growth stages. In order to reveal the correlation between dominant bacteria units as biomarkers and population dynamics in rotifer, we conducted five times repeated 10-fold cross-validation to evaluate the importance of bacterial taxa, and screened 36 OTUs as biomarkers in the model (Supplementary Figure S1). The relative abundance of the first 36 biomarker bacteria units is variable at different growth stages in *B. calyciflorus*. For example, OTU150 (Comamonadaceae), OTU3708 and OTU2725 (Cyanobiaceae) kept a high level in the whole lifespan (T1-T4), and OTU536 (Flavobacteriaceae) increased significantly in the slow growth period (T2), while OTU3635 (Candidatus Hepatincola) had a high relative abundance in both exponential growth and decline periods (Figure 8).

## 4. Discussion

### 4.1. Effect of associated bacteria on the growth and fecundity of *Brachionus calyciflorus*

In ecological food webs, bacterial transformation is considered to be an important pathway of the carbon and nitrogen cycle (Azam and Malfatti, 2007; James et al., 2017). The intestinal tract and outer surface of zooplankton can be good colonization conditions for bacteria (Tang et al., 2011), while the attached benign bacteria may also have a probiotic effect and promote the adaptability of the host to the environment (Verschuere et al., 2000), for example, the change of bacterial community structure can regulate the tolerance of zooplankton to toxic cyanobacteria (Macke et al., 2017). In most of the past studies, single or mixed probiotics were added to explore the effect of bacteria on rotifer reproduction from the perspective of diet (Sarma et al., 2001; Devetter and Sed'a, 2003), while the present experiment simulated the habitat bacterial environment to explore the impact of bacterial community itself on the life history strategy and population growth of *B. calyciflorus* at the same dietary conditions. In iterative organisms including rotifers, the life history information collected by life table and population growth research are considered to be complementary (Sarma et al., 2006). According to the results of the life table, the change of physical and chemical factors in the culture media had no significant impact on the life expectancy (*e*_0_), and the bacteria alone did not affect rotifer fecundity, while the bacteria cultured in the nature water (*in situ*) significantly increased rotifer reproduction rates (*R*_0_ and *r*_m_). However, compared with the sterile EPA treatment (SE), the generation time and maxis ratio of *B. calyciflorus* had no significant change in the other two groups, which was contrary to the results from Hagiwara et al. (1994) that the addition of bacteria in culture can promote the sexual reproduction of rotifers under the same conditions. At the same time, the results of the population growth experiment confirmed that associated bacteria community in nature water can enhance the population growth rate (*r*) and maximum density in *B. calyciflorus*, which is consistent with the findings by Contreras-Tapia et al. (2020a) where rotifer growth rates and maximum densities were higher in the treatments with probiotics and algal food compared to the control with only algal food (probiotic-free). It is thus evident whether bacteria added as pure free live (Le et al., 2017), or added as a mixture of laboratory-cultured or commercial products (Douillet, 2000; Contreras-Tapia et al., 2020b), both possibly improve the growth and fecundity performance of rotifers. Furthermore, these results also suggest that the associated bacterial community carried within the resting eggs does not sufficient for the optimal growth of rotifers and still needs to be replenished from outside, such as from bacterioplankton in the surrounding water.

The *T. obliquus* in this study served as food, which itself carried some bacteria. The relative abundance of *Hydrogenophaga* was high among the *T. obliquus*-associated bacteria, and it was also prominent among the *B. calyciflorus*-associated bacteria. In contrast, *Brevundimonas* had the highest relative abundance among the *T. obliquus*-associated bacteria, but was not prominent among the *B. calyciflorus*-associated bacteria. On the one hand, those results show that not all of the bacteria associated with algae are favorable for the growth of rotifers and are equally inadequate for the optimal growth of rotifers, so the supplementation of rotifer-associated communities with probiotic bacteria from outside is still necessary (Le et al., 2017; Contreras-Tapia et al., 2020b). On the other hand, the rotifer-associated bacterial community detected included a partially associated bacteria of algae and inside resting eggs, but we did not remove sequences shared with algae-associated bacteria from the rotifer-associated bacteria, as we were concerned that might underestimate the importance of those bacteria. The present study focuses on the importance of supplementing external bacterioplankton for rotifer growth and reproduction, but the bacteria with true intrinsic symbiotic of rotifer will be of equal interest to us in the future.

### 4.2. Flexibility and keystone taxa of *Brachionus calyciflorus*-associated bacteria

It has been shown that the composition of bacterial community associated with freshwater zooplankton is very flexible, and is greatly influenced by the environment through active exchanges with habitat bacteria community (Eckert et al., 2021), and most of them come from the surrounding water environment (Eckert et al., 2020). In this study, compared with sterile culture, the diversity of the combined bacteria in *B. calyciflorus* increased significantly when cultured in the SEB and NW media. In addition, the similarity between SEB and NW bacterial communities associated with rotifers compared to SE probably suggest that the microbiome of the rotifers is strongly influenced by the bacteria present in the environment. The present results suggested that Proteobacteria is the main associated bacteria with *B. calyciflorus*, followed by Bacteroidota, Actinobacteriota, Cyanobacteria and Firmicutes. Recent studies have also found that Proteobacteria dominate the bacterial community associated with rotifers (Turgay et al., 2020). Different from the results of this study, Actinobacteriota is the dominant taxa associated with rotifers in another culture-dependent experiment (Ishino et al., 2012). Meanwhile, the Firmicutes had high relative abundance in all treatment groups, which indicates that there may be good anaerobic conditions in the rotifer intestine (Shoemaker and Moisander, 2015).

In this experiment, 482 OTUs from the *B. calyciflorus*-associated bacteria were shared by three culture groups, accounting for about 15% of the relative abundance, however significant differences in bacteria diversity and community composition occurred among different treatments. For example, unclassified Alphaproteobacteria OTUs, Rhizobiaceae OTUs and Moraxellaceae OTUs had higher relative abundance in the dominant taxa in all treatment groups, while the relative abundance of Comamonadaceae OTUs and Flavobacteriaceae OTUs in the SE group was higher than that SEB and NW groups. It has been suggested that Comamonadaceae is the main bacterial group in the intestinal microflora of cladocera and rotifers, which can improve the adaptability of zooplankton to the environment (Freese and Schink, 2011; Akbar et al., 2021). The Flavobacteriaceae was also an important taxa in the copepod-associated bacterial community and a member of fish intestinal microflora (Corte et al., 2018; Egerton et al., 2018). Members of this bacterial branch can degrade high molecular weight organic matter, such as cellulose and chitin, indicating symbiotic or parasitic interaction between *Flavobacterium* and zooplankton (Beier and Bertilsson, 2013). The molts and carcasses of zooplankton are also the main sources of chitin in waters, and the colonization by bacteria may also play a key role in the carbon and nitrogen cycle in the aquatic ecosystem (Tang et al., 2010). Studies have shown that the bacterial community composition associated with rotifers is highly flexible due to the influence of diet, habitat bacterial community and environmental factors (Turgay et al., 2020), in consideration of the understanding of rotifer-associated bacterial community is very limited at present, thus it is inaccurate to determine the core taxa only from the relative abundance, and the significance and proportion of the underlying key taxa could be underestimated. In addition, only one natural water source was studied in this paper, and the scope of the study needs to be expanded in the future as a way to enhance the accuracy of the core flora study.

A correlation network is a new means that is often used to speculate and identify keystone taxa of microorganisms (Si et al., 2017). The keystone taxa may play an important role in some processes such as nutrient cycling and energy flow, thus influencing the ecological functions of bacterial communities (Fierer, 2017). The higher the complexity of the network structure, the greater the number of key taxa it requires (Banerjee et al., 2019). In this study, the correlation network was selected to speculate the keystone taxa of *B. calyciflorus*-associated bacteria. The results demonstrated there were more keystone taxa of *B. calyciflorus*-associated bacteria in the NW group, and the growth status of the rotifer population was better. Among the three treated groups, the relative abundance of Burkholderiales and Pseudomonadales was higher, and numerous members of these two orders have been consistently identified as keystone taxa in different studies and different ecosystems (Banerjee et al., 2018). In addition, Environmental water can become contaminated from a variety of sources, such as human activities (Staley et al., 2012), therefore *Bifidobacterium* was found in rotifer culture and may play a beneficial role as they accumulate in rotifer culture (Samat et al., 2020). In addition to these well-known bacteria, some keystone taxa with low relative abundance, such as Caulobacterales (OTU493), Microtrichales (OTU754), and Solirubrobacterales (OTU580), were identified in this study. The keystone taxa may play an important role in some processes such as nutrient cycling and energy flow, thus influencing the ecological functions of bacterial communities, but are not linked to their relative abundance (Fierer, 2017). On the other side, some studies have indicated that keystone taxa may not change with environmental fluctuation and should always be present (Vandenkoornhuyse et al., 2015), while Banerjee et al. (2018) held that members of keystone taxa may be functionally redundant and their membership is not set in stone. Although there have been previous studies exploring core taxa in the associated bacteria with zooplankton (Shoemaker and Moisander, 2015; Szokoli et al., 2016), there are very limited reports on keystone taxa in the associated bacteria of *B. calyciflorus*. This work was implemented to identify keystone taxa from the correlation of bacterial network structure. For further exploration, the influence of excluding keystone taxa on the function and composition of the overall bacterial community is necessary to follow, and it is also worth the effort to evaluate the importance of rare taxa to symbiotic organisms.

### 4.3. The associated bacterial groups and biomarkers related to population dynamics of *Brachionus calyciflorus*

Host-related bacterial communities are usually obtained through the horizontal transmission of bacteria existing in the environment, so the difference in habitat bacterial community will affect the assembly of bacteria on the host (Callens et al., 2018). Simultaneously, the host is also selective to different bacterial communities (Tang, 2005), and the composition of host-associated bacteria will vary with the change of growth conditions and stages, which will affect the adaptability of the host and promote or inhibit vital activity (Sakami et al., 2014; Peerakietkhajorn et al., 2016; Macke et al., 2017).

In the present study, the relative abundance of 7 genera of *B. calyciflorus*-associated bacteria increased significantly during slow population growth and exponential growth. The genera *Hydrogenophaga*, unclassified Comamonadaceae and *Acidovorax* in Comamonadaceae, *Flavobacterium* in Flavobacteriaceae, and *Rheinheimera* in Alteromonadaceae are closely related to denitrification (Calderer et al., 2014; Lv et al., 2017; Deng et al., 2020). It is reported that *Acidovorax* can promote the growth of *Daphnia magna* (Akbar et al., 2021), and *Flavobacterium* and *Rheinheimera* participate in the degradation of chitin and other polysaccharides, which is an important way of carbon and nutrient cycle (Wietz et al., 2015). In general, zooplankton release nutrients or secrete organic compounds through excretion or body decomposition, which provides a good nitrogen source and carbon source for denitrifying bacteria (Corte et al., 2018), therefore the change of denitrifying bacteria community structure in response to the population dynamics of zooplankton mediated by the alteration of ammonia nitrogen, total nitrogen and nitrate nitrogen (Lu et al., 2016). On the other hand, these functional bacteria also provide important elemental nutrient sources for zooplankton and maintain the ecological balance of carbon and nitrogen, so the increase in the relative abundance of these bacteria may improve the population growth of zooplankton (Martin-Creuzburg et al., 2011). *Novosphingobium* is a member of Sphingomonadaceae, which widely exists in water, soil and other environments (Jin et al., 2019), also in the microflora of organisms such as *Actinidia deliciosa* (Wu et al., 2019), *Aedes aegypti* in Singapore (Macleod et al., 2021) and humans (D’Auria et al., 2013). *Novosphingobium* members have the ability to biodegradation, such as degrading microcystins for regulating the tolerance of zooplankton to toxic cyanobacteria in eutrophic lakes (Wang et al., 2019). *Limnobacter* is a thiosulfate-oxidizing bacterium, which participates in sulfur oxidation, citric acid cycle, phenol degradation and various organic matter transport processes (Vedler et al., 2013; Xiao et al., 2016), whose members are components of copepod-associated bacteria (Sadaiappan et al., 2021), also present in the skin of zebrafish (*Danio rerio*) (Coetzer et al., 2021).

The relative abundance of another dominant bacterium associated with *B. calyciflorus* decreased significantly during the slow and exponential periods of population growth. *Elizabethkingia* is a gram-negative bacillus that exists in rivers, reservoirs and other water sources (Reche et al., 2016). It has also been found many times in humans and animals like fish (Laith et al., 2017) and frogs (Trimpert et al., 2021). The members of *Elizabethkingia* are pathogenic and will pose a threat to human life, especially in premature newborns and immunocompromised people (Baruah et al., 2020). Chitin has been reported to inhibit the growth of some pathogenic bacteria (Lopez-Santamarina et al., 2020). Chitin is abundant in the exoskeleton of rotifers (Tang et al., 2010), and its accumulation in rotifer culture with increasing population density could account for the significant decrease in the relative abundance of the genus *Elizabethkingia*.

Among the dominant taxa of *B. calyciflorus*-associated bacteria, unclassified Alphaproteobacteria and A*llorhizobium-Neorhizobium-Pararhizobium-Rhizobium* of Rhizobiales were the two genera with the highest relative abundance, which have no obvious changing trend during the whole population growth cycle. Studies have shown that Alphaproteobacteria is not only dominant in rotifer-associated bacteria, but also widely exists in Cladocera (Grossart et al., 2009), Copepods (Dziallas et al., 2013) and Protozoa (Khalzov et al., 2021). The members of Rhizobiales are mostly nitrogen-fixing bacteria, which are the most common plant symbioses and also play a potential role in the carbon cycle of freshwater lakes (Erlacher et al., 2015; Wang et al., 2021).

Using a stochastic forest model, we further identified the types of biomarkers associated with population density changes in *B. calyciflorus*. In addition to OTU150 (Comamonadaceae) and OTU536 (Flavobacteriaceae) which may participate in the process of nutrient cycle and promote the increase of population density in *B. calyciflorus*, OTU3708 and OTU2725 belonging to Cyanobiaceae also have the function of nitrogen fixation and are important primary producers in the aquatic ecosystem. Studies have shown that the growth and reproduction of zooplankton such as Cladocera and rotifer are positively correlated with cyanobacteria (Jia et al., 2017; Motwani et al., 2018). However, the OTU3708 kept a high level in the whole growth cycle (T1-T4) in this study (SEB and NW), which had no obvious correlation with rotifer population density, coinciding with the findings of Suikkanen et al. (2021). The OTU3635 belongs to *Rickettsia*, which is an exclusive intracellular parasite and can infect almost all species of major eukaryotic lineage (Kang et al., 2014), such as ciliates (Szokoli et al., 2016), and *B. calyciflorus* may also serve as its host.

## 5. Conclusion

In this study, it was found that the reconstruction of the *B. calyciflorus*-associated bacterial community can increase the diversity of the bacterial community, and promote the population growth and fecundity of *B. calyciflorus*, resulting in the enhancement of its life expectancy at hatching, net reproduction rate, intrinsic growth rate, population growth rate and maximum population density, but no significant effects were detected on the generation time and sexual reproduction. The change of physicochemical factors in the culture media had no significant impact on the life expectancy, and the bacteria alone did not affect rotifer fecundity, while the bacteria cultured in the nature water (*in situ*) significantly increased rotifer reproduction rates. In addition, whether the *B. calyciflorus*-associated bacteria community was reconstructed or not, it is mainly composed of Proteobacteria, Bacteroidota, Actinobacteriota, Cyanobacteria and Firmicutes. A correlation network analysis revealed that some members of Burkholderiales, Pseudomonadales, Micrococcales, Caulobacterales and Bifidobacteriales may be the keystone taxa of *B. calyciflorus*-associated bacteria. The relative abundance of these bacteria increased obviously during the slow and exponential periods of population growth. Meanwhile, it was found that the *B. calyciflorus*-associated bacteria also contained some pathogenic bacteria or parasitic bacteria, such as *Elizabethkingia* and *Rickettsiales*, and their relative abundance decreased significantly with the increase of rotifer population density. In the present study, we explored the effect of reconstructing the associated bacterial community from natural water on the population dynamics of *B. calyciflorus*, but the associated bacteria composition is flexible and greatly influenced by the surrounding waters, hence it is necessary to further explore the influence of keystone taxa and rare taxa on the association between rotifers and bacterioplankton.

## Data availability statement

The datasets presented in this study can be found in online repositories. The names of the repository/repositories and accession number(s) can be found at: https://ngdc.cncb.ac.cn/gsa, CRA007441.

## Author contributions

XX: conceptualization and funding acquisition. YZ: methodology, data curation, formal analysis, writing—original draft preparation, and writing—review and editing. SF: formal analysis and writing—original draft preparation. LZ: writing—original draft preparation. ML: writing—original draft preparation. All authors reviewed the manuscript.

## Funding

This work was supported by the Natural Science Foundation of China under Grant (31872208), and the University Synergy Innovation Program of Anhui Province under Grant (GXXT-2020-075).

## Conflict of interest

The authors declare that the research was conducted in the absence of any commercial or financial relationships that could be construed as a potential conflict of interest.

## Publisher’s note

All claims expressed in this article are solely those of the authors and do not necessarily represent those of their affiliated organizations, or those of the publisher, the editors and the reviewers. Any product that may be evaluated in this article, or claim that may be made by its manufacturer, is not guaranteed or endorsed by the publisher.

## Footnotes

^1^http://www.majorbio.com/

^2^http://www.drive5.com/-uparse/

^3^https://www.arbsilva.de/

^4^ https://sourceforge.net/projects/rdpc-lassifier/

^5^ http://qiime.org/install/index.html
