# Supplementary material for: Population dynamics of Brachionus calyciflorus driven by the associated natural bacterioplankton
Source: Front Microbiol. 2023 Jan 16;13:1076620. doi: 10.3389/fmicb.2022.1076620 (PMC9884981; doi:10.3389/fmicb.2022.1076620)
Supplement: Supplementary file 2 [file Data_Sheet_1.PDF]

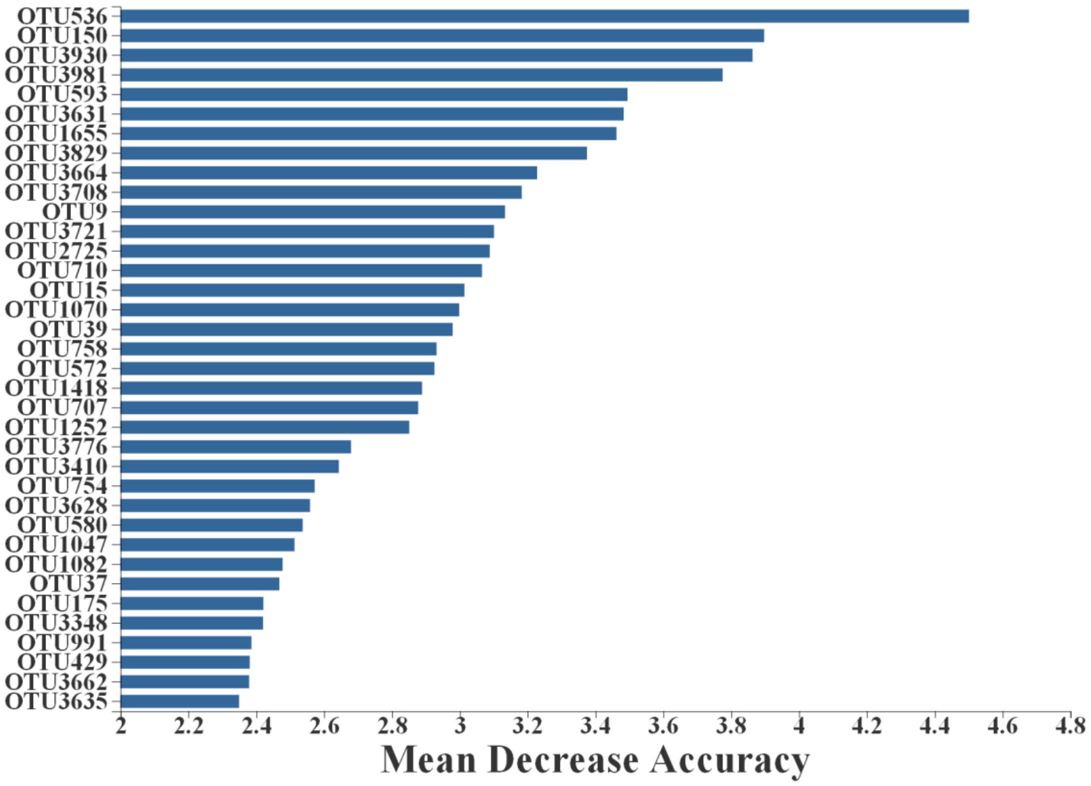

Figure S1 The importance ranking of top 36 dominant OTUs in associated bacteria community assessed by Mean Decrease Accuracy (based on 500 decision trees). The X axis is equal to the measured value/standard difference of species importance, and the Y axis corresponds to the species names sorted by importance.
